# The mouth or the nose: the past, present, and future of ultra-slim gastroscopy of the upper gastrointestinal tract in pediatrics

**DOI:** 10.3389/fped.2025.1630157

**Published:** 2025-07-22

**Authors:** Paul Tran, Rose Lee, Ali Mencin, Matthew Ryan, Joel A. Friedlander, Michael A. Manfredi

**Affiliations:** ^1^ Division of Pediatric Gastroenterology, Phoenix Children's Hospital, Phoenix, AZ, United States; ^2^ Division of Pediatric Gastroenterology, The Medical College of Wisconsin, Wisconsin, WI, United States; ^3^ Division of Pediatric Gastroenterology, Columbia University Vagelos College of Physicians and Surgeons, New York, NY, United States; ^4^ Division of Gastroenterology, Hepatology and Nutrition, Children's Hospital of Philadelphia, Philadelphia, PA, United States; ^5^ EvoEndo, Inc., Centennial, CO, United States

**Keywords:** pediatrics, gastroenterology, ultra-slim, ultrathin, endoscopy, transnasal endoscopy

## Abstract

**Background:**

Ultra-slim, flexible endoscopy pioneered for the evaluation of luminal diseases of the airway, has been used for over 40 years. In the late 20th century, it was adopted in the gastrointestinal specialties for evaluation of the digestive tract with and without the need for sedation. Since the advent of this technology, numerous descriptions of its use in small anatomic areas have been described. These include stricture evaluation and therapy, ostomy evaluation, biliary interventions, and diagnostic endoscopic evaluation of pediatric patients. This manuscript reviews the availability and clinical utility of ultra-slim flexible endoscopes, describes technical approaches, and highlights the potential value the technology offers to pediatric providers and healthcare systems.

**Methods:**

A comprehensive literature review was conducted on the use of ultra-slim endoscopes in the pediatric and adult gastroenterology. Expert discussions were held to evaluate current practices, indications, and equipment. The resulting expert opinion was generated to summarize pertinent information regarding key techniques, indications, and practical considerations for implementation in pediatric settings.

**Results:**

Although ultra-slim endoscopes started their use in small-space adult gastroenterology procedures, they have shown significant benefit and value within pediatrics. The literature documents their use in both sedated and unsedated environments, including transnasal upper endoscopy, variceal surveillance, esophageal stricture evaluation, neonatal endoscopy, enteral tube placement, and ostomy assessment.

**Conclusion:**

Ultra-slim flexible endoscopy provides pediatric gastroenterologists with a valuable diagnostic and therapeutic tool. When used appropriately, either with or without sedation, it has the potential to increase clinical efficiency, reduce procedural risk, and improve access to care.

## Introduction

Ultra-slim, sedation-free, flexible endoscopy of the entire gastrointestinal tract was first reported in 1994 by Dr. Reza Shaker of the Medical College of Wisconsin as he was pursuing a novel way to investigate gastroesophageal reflux in adults (1). He was part of a group that previously evaluated the relationship between the esophagus, glottis and pharynx using a technique similar to otolaryngologists that utilized topical analgesia to perform transnasal flexible endoscopy using bronchoscopes (2–5). By 1994 rapidly advancing technology enabled his group to evaluate beyond the esophagus and proximal stomach (1). Subsequent to this original manuscript numerous publications discussing unsedated transnasal endoscopy (TNE) using ultrathin, flexible, long, endoscopes documented the technique's success, safety, patient preference, and utility across a spectrum a conditions such as Barrett's Esophagus, gastroesophageal reflux disease (GERD), and esophageal varices (1, 6–11). Though the technique in adults was found to be highly successful, adoption in the United States remained poor (12). This was hypothesized to be due to a variety of economic and training factors (11–13). Globally it was found to have better traction, but its use was still low outside the Asian continent (12). By 2010, the slim, 5–6 mm outer diameter (OD), gastroscopes utilized for the technique (standard endoscopes: 8.6–10 mm OD) also found other uses beyond the original sedation-free/unsedated technique described by Shaker (10). This was noted in a consensus document by the American Society of Gastrointestinal Endoscopy and included sedated uses in small children and narrow areas of the gastrointestinal tract (10).

By 2016 unsedated TNE evaluating the gastrointestinal tract continued to be poorly adopted by adult gastroenterologists in the United States, but trends started to change as new procedural coding and publications were seen from otolaryngologists, physician extenders, and pediatric gastroenterologists (14–16). The first series of sedation-free transnasal GI tract evaluation in pediatrics appeared in publication from an aerodigestive group that reported its use to monitor eosinophilic esophagitis (EoE) (14). It was studied in this population because of the surge of the endoscopy for EoE being performed in children along with the Food and Drug Administration (FDA) issuing warning regarding the repetitive use of anesthesia in children (17). Strangely enough the pediatric technique was also studied using bronchoscopes, but by this time the OD of bronchoscopes was much smaller with reports of 2.8–4.0 mm used depending on the size of the child (14). Evaluation of the biopsies for EoE found adequate tissue specimens were obtained regardless of the biopsy tool size (18–20). After this initial study, numerous subsequent articles were published documenting the unsedated technique's use for esophageal varices, celiac disease, esophagitis dessicans, celiac disease, esophageal atresia (EA), disease, Barrett's esophagus, and H. Pylori (18, 21–27). Newer studies in pediatrics also further demonstrated the potential utility of the narrow scopes for unsedated ostomy endoscopy, their use in a sedated environment, or even for therapeutic endoscopy (28, 29). With burgeoning interest in the use of ultra-slim endoscopy, in 2022, a new slimmer, 3.5 mm, single-use, ultra-slim gastroscope was released for use the pediatric and adult population. Subsequent evaluations of its uses in various contexts have been reported (26–28).

This manuscript reports on the available literature on the use of ultra-slim and ultrathin endoscopes and expands on it with expert opinion on their use in a variety of sedation-free and sedated environments. It hypothesizes concepts and reports on both utility and limitations in each setting.

## Section 1: technical aspects of slim gastroscopes, flexible endoscopes, and equipment

The basic design of slim, reusable gastroscopes is similar across all models and consists of three main parts: the control section, the insertion tube, and the connector section. The control section, intended to be held in the left hand, has two stacked control dials that maneuver the instrument tip. One dial deflects the tip up and down, and the other deflects it left and right. Both dials can be locked into place for prolonged tip deflection. The control section also includes separate buttons for electronic functions, suction control, and air/water insufflation. Other specialty ultra-slim endoscopes usually have a variant control section. These include a steering lever (rather than dials), a single mechanical button for a single function, and electronic function buttons.

The insertion tube is a flexible shaft attached to the control section. It contains a lens wash channel as well as a working channel that accommodates accessory passage and enables suction and insufflation. In slim gastroscopes, the channel diameter typically ranges from 2.0–2.4 mm. The insertion tube also houses angulation wires that facilitate deflection of the instrument tip. At the tip of a video endoscope, there is a charge-coupled device (CCD) or complementary-metal-oxide-semiconductor (CMOS) for color image generation, a light illumination system, and an opening for the air/water channel. Table 1 outlines the key technical aspects of various flexible, ultra-slim endoscopes.

**Table 1:** Partial listing of ultra slim flexible gastroscopes and bronchoscopes available.

Manufacturer^a^	Scope Type	Scope Model #	Insertion Tube Diameter shaft/tip(mm)	Insertion Tube length (cm)	Working Channel Diameter (mm)	Field of view (^○^)	Bending capability up/down (^○^)	Bending capability (left/right)
Ambu	Single Use Bronchoscope	Ambu® aScope™ 5 Broncho 2.7/1.2	2.7/3.0	60	1.2	120	210/210	120 (using rotary function)
	Single Use Bronchoscope	Ambu® aScope™ 5 Broncho 4.2/2.2	4.2/4.4	60	2.2	120	210/210	120 (using rotary function)
	Single Use Bronchoscope	Ambu® aScope™ 5 Broncho 5.0/2.2	5.0/5.4	60	2.2	120	195/195	120 (using rotary function)
	Single Use Bronchoscope	Ambu® aScope™ 4 Broncho Slim	3.8/4.2	60	1.2	85	180/180	NA
	Single Use Bronchoscope	Ambu® aScope™ 4 Broncho Regular	5.0/5.4	60	2.2	85	180/180	NA
Boston Scientific	Single Use Bronchoscope	EXALT Model B Slim	4.3/3.8	60	1.0	90	180/180	NA
	Single Use Bronchoscope	EXALT Model B Regular	5.5/5.0	60	2.0	90	180/180	NA
EvoEndo	Single use Gastroscope	LE 110	3.5/3.5	110	2.0	120	210/90	180/180
	Single use Gastroscope	LE 85	3.5/3.5	85	2.0	120	210/90	180/180
Fujifilm	Reusable Gastroscope	EG-740N	5.9/5.8	110	2.4	140	210/90	100/100
	Reusable Bronchoscope	EB-710P	4.1/4.1	60	2.0	120	210/130	120 (using rotary function)
	Reusable Bronchoscope	EB-580S	5.1/5.3	60	2.2	120	210/130	120 (using rotary function)
	Reusable Bronchoscope	EB-530P	3.8/3.8	60	1.2	120	180/130	120 (using rotary function)
	Reusable Bronchoscope	EB-530S	4.9/4.9	60	2.0	120	180/130	120 (using rotary function)
	Single Use Bronchoscope	SBV-1A-B	2.8/2.8	60	1.2	120	220/220	60 (using rotary function)
	Single Use Bronchoscope	SBV-1B-B	4.2/4.2	60	2.0	120	220/220	60 (using rotary function)
	Single Use Bronchoscope	BCV1-S2	5.8/5.7	60	2.8	110	210/210	90 (using rotary function)
Olympus	Reusable Gastroscope	GIF-XP190N	5.8/5.4	110	2.2	140	210/90	100/100
	Reusable Bronchoscope	BF-Q190	4.9/4.8	60	2.0	120	210/130	120 (using rotary function)
	Reusable Bronchoscope	BF-H190	5.1/5.5	60	2.0	120	210/130	120 (using rotary function)
	Reusable Bronchoscope	BF-P190	4.1/4.2	60	2.0	110	210/130	120 (using rotary function)
	Reusable Bronchoscope	BF-MP190F	3.7/3.0	60	1.7	90	210/130	120 (using rotary function)
	Reusable Bronchoscope	BF-XP190	2.8/3.1	60	1.2	110	210/130	120 (using rotary function)
	Single Use Bronchoscope	BCV1-C2	3.2/3.3	60	1.2	110	210/210	90 (using rotary function)
	Single Use Bronchoscope	BCV1-M2	4.9/4.8	60	2.2	110	210/210	90 (using rotary function)
Pentax	Reusable Gastroscope	EG-1690K	5.4/5.4	110	2.0	120	210/120	120/120
	Reusable Bronchoscope	EB11-J10	3.9/3.7	60	1.2	120	210/130	NA
	Reusable Bronchoscope	EB15-J10	5.2/5.4	60	2.0	120	210/130	NA
Verathon	Single Use Bronchoscope	BFlex 2	3.8/3.8	60	1.2	120	180/185	NA
		Slim 3.8						
	Single Use Bronchoscope	BFlex 2	5.0/5.0	60	2.2	120	215/225	NA
		Reg 5.0						

^a^ Although the authors have tried to be as complete as possible, this list may not include all slim gastroscopes or bronchoscopes.

A newly released single-use ultra-slim gastroscope (EvoEndo Scope, Inc, Grayslake, IL) has a slightly different design. Its controller differs from that of traditional reusable gastroscopes. Instead of a wheel for up/down deflection, the Model LE has a central control lever similar to a bronchoscope, while two wheels on either side regulate left/right deflection. Rather than the traditional two buttons, there are three buttons that respectively control air, water, and suction. The insertion tube comes in two lengths (85 cm and 110 cm), has an outer diameter of 3.5 mm, and a 2.0 mm working channel. There is no separate lens wash channel. See Table 1 for technical aspect of this gastroscope.

Historically, slim endoscopes have primarily been used for diagnostic purposes, and multiple companies offer biopsy forceps designed to fit a 2.0 mm working channel (10). Although the range of therapeutic disposable devices for this channel size is more limited than for the 2.8 mm channel, a variety of tools are still available. Certain manufacturers produce smaller-gauge injection needles suited for localized medication delivery, while polypectomy snares adapted for slim gastroscopes facilitate the removal of polyps and assist in foreign body retrieval. Specialized retrieval instruments, such as grasping forceps and nets, further enhance the scope's utility by enabling efficient extraction of objects from pediatric patients.

A selection of cautery and hemostasis devices are also compatible with some of the ultra-slim gastroscopes. Straight-fire argon plasma coagulation (APC) catheters are on the market, and at least one company offers a bipolar probe that fits the 2.0 mm channel. Needle knives designed for slim gastroscopes support incisional therapy when needed. Although the selection of devices is narrower than for standard endoscopes, these options support a wide range of therapeutic interventions. Please see Table 2 for a list of compatible devices.

**Table 2:** List of various therapeutic devices that can fit into a smaller 2.0 mm working channel to be used for reference but may not include every existing device.

Single Use Therapeutic Devices That Can Be Used In A 2.0 Mm Working Channel^a^			
Device Type	Device Name	Manufacturer	Comments
Injection Needle	Interject™	Boston Scientific	Working length: 200 cm
			Needle Gauge 23 and 25 Gague,
		(Marlborough, MA)	Needle length 4 and 6 mm
	Click-Tip™	CONMED	Working Length 230 cm
		(Largo, FL)	Needle Gauge 22 and 25 Gauge
			Needle length 4 and 6 mm
	Disposable Varices Injector	Cook Medical (Bloomington, IN)	Working length: 200 cm
			Needle Gauge 23 and 25 Gague,
			Needle length 4 mm
	Posi-Stop Injection Needles	HOBBS Medical (Stafford Springs, CT)	Working Length 165 cm
			Needle Gauge 23 and 25 Gauge,
			Needle length 5 mm
	Injector Force Max	Olympus (Center Valley, Pa)	Working Length 165 cm
			Needle Gauge 23 and 25 Gauge,
			Needle length 4, 5, and 6mm
	CARR-LOCKE Injection Needle	Steris (Mentor, OH)	Working Length 230 cm
			Needle Gauge 25 Gauge
			Needle length 5 mm
	Sure-Stop Sclerotherapy Needle	TeleMed Systems (Hudson, Mass)	Working Length 200 cm
			Needle Gauge 23 and 25 Gauge,
			Needle length 5 mm
Polyp Snare	Profile™ Single-Use Snares	Boston Scientific	Working length: 240 cm
			Opening width: 11, 13, and 27 mm
	SnareMaster™	Olympus	2.0 available only in Single-use crescent shape
	Pediatric Snare	TeleMed Systems	Working length: 180, 240 cm
			Opening width: 25 mm
Bipolar Probes	BiCap® Superconductor™	CONMED	Tip length 6.5 mm
			Working length: 200 CM
Needle Knife	Huibregtse® Single Lumen Needle Knife	Cook Medical	Working Length 200 cm
			Needle length 4 mm
			Used for EIT
Retrieval Grasper	Rescue Rat Tooth	Boston Scientific	Working length 180 cm
			Opening width 4.5 mm
	Caesar® Grasping Forceps	Cook Medical	Working length 240 cm
			Three prong grasper
	PolyGrab- Single-use grasping forceps—two-prong	Olympus	Working length: 155 cm
			Opening width 14 mm
	PolyGrab™: tripod mini grasping forceps	Olympus	Working length 115 cm
			Opening width 10 mm
	Rat tooth	Olympus	Working length 190 cm
			Opening width 3.8 mm
	grasping forceps—rubber tips	Olympus	Working length 190 cm
			Opening width 4.8 mm
	Raptor grasping device—mini	Steris	Working length 200 cm
			Opening width 7 mm
	Four prong Grasping Forceps	TeleMed Systems	Working length 180 cm
			Opening width: 25 mm
Retrieval Net	SwirlNet®	Olympus	Working length 160 cm
			Net specs 2 cm x 4.5 cm
	Roth Net® Retriever—Mini	Steris	Working length 160 cm
APC probes	Beamer AVEO™	CONMED	Working length 160 cm
	FiAPC® probe	ERBE (Marietta, GA)	Working length 150 cm
	APC Axial Probe	Olympus	Working length 300 cm

^a^ Although the authors have tried to be as complete as possible, this list may not include all single-use devices available from various manufacturers.

## Section 2: traditional sedation-free transnasal endoscopy (TNE) as transnasal esophagoscopy (TN-ESO), transnasal esophagogastroscopy (TN-EG) and esophagogastroduodenoscopy (TN-EGD)

Sedation-free TNE is a more cost-effective, safe, less invasive, and better-tolerated diagnostic method for evaluating the esophagus, stomach, and duodenum compared to sedated upper gastrointestinal (GI) endoscopy (7, 18, 30, 31). It is employed as a diagnostic tool in the evaluation of the gastrointestinal tract or as a surveillance tool for children with eosinophilic esophagitis or other esophageal conditions who need ongoing endoscopic monitoring (18, 23, 30, 32, 33). Indications for sedation-free TNE in pediatrics have included evaluating abdominal pain, dysphagia, esophageal atresia/tracheal esophageal fistula odynophagia, acid reflux, Barrett's esophagus, celiac disease, H. Pylori, and emesis (18, 25). Relative contraindications to consider include abnormal nasal cavity, severe anxiety, recent significant illness (< a week) such as sepsis, coagulopathy, frequent epistaxis, significant developmental delay, or a history of aspiration.

Following the U.S. Food and Drug Administration's (FDA) warning about the potential effects of repeated general anesthesia on brain development in young children, parents have become increasingly interested in sedation-free endoscopy (34). The original reports noted use of off-label ultra-slim flexible bronchoscopes, but in 2022, a single-use flexible gastroscope (EvoEndo, Inc, Grayslake, IL) was cleared by the FDA for use in children aged 5 years and older (14, 27). Over the years, pediatric gastroenterologists have developed various techniques to make sedation-free TNE more comfortable for children (18). This report presents the authors' experience and recommendations regarding the method of performing transnasal endoscopy (TNE) without sedation.

Prior to the procedure being performed, the provider needs to find appropriate patients and have a discussion with the family about the procedure. This usually occurs at an office visit similar to how any other form of endoscopy is discussed. The authors have found that individuals >5 years of age who have had a successful nasal procedure (laryngoscopy, nasogastric tubes), blood draw, or basic medical procedures are usually great candidates. When first learning TNE the larger anatomy associated with age-appropriate sized patients >8 years are often ideal. Sometimes 8–12-year-old patients are more cooperative than teenagers, depending on personality. High functioning children with autism are often found to be highly successful with TNE as well.

When offering the procedure to a potential patient and family the conversation is of utmost importance. Education around how to have this conversation is important for the endoscopist as well as for the endoscopist's colleagues. Though TNE has been reported to be highly successful in both adult and pediatric populations, many physicians struggle with how to discuss, offer, or recommend sedation-free procedures to a family, especially when first starting. This may create minimal procedural opportunities for the endoscopist and team's staff to become proficient. This group suggests the physician approach the family and patient using a shared decision-making paradigm. Specifically in this case, this would include a physician or provider outlining the procedure's clinical benefits over sedated endoscopy, offering a clear and confident recommendation, and alleviating concerns of the family/patient. This is followed by a discussion with the family and mutually deciding on the best procedural option. This should be done without diminishing the success or safety of sedated endoscopy. For example, a physician may highlight the benefits of sedation-free endoscopy that include an increased safety profile by eliminating anesthesia, the lack of need for needles, less time away from activities, less time fasting, opportunity to use virtual reality distraction, family involvement, and often easier and quicker access to diagnosis and surveillance. They would also confidently discuss the procedure's success across age ranges, specifically in the pediatric population. Showing the family videos, the equipment or handouts, or even the equipment/room can also be very helpful. If the opportunity presents itself discussing the procedure before it is going to be needed may offer the family more time to think about procedural options. The authors also suggest noting to the family that if the sedation-free procedure doesn't go well or doesn't work, sedated endoscopy is an option in the future. A recommendation to try the unsedated procedure as an initial trial is often very helpful as well. Studies of patient perspectives after TNE show that they will still opt for the sedation-free procedure, even if they still have reservations (14, 18, 35). For a full review of patients perspectives on the procedure Scherer et al. discussed the parental perspectives on the technique in 2021 and confirmed what parents found most beneficial (35).

Proper procedural preparation prior to the procedure day is also important. After the provider recommends the procedure but before the endoscopy visit, the child and family are prepared, typically by a member of the medical team (physician, nurse, assistant, etc.). This preparation usually involves a discussion and/or review of video materials about the procedure. The same individual often asks screening questions (e.g., regarding significant illness) and provides Nil Per Os (NPO) guidelines. For Transnasal Esophagoscopy (TN-Eso), the NPO guidelines are typically 2 h, with a third hour allowing no more than 2 ounces of clear liquid. For Transnasal Esophagogastroscopy (TN-EG) or Transnasal Esophagogastroduodenoscopy (TN-EGD), the NPO period is usually 4–6 h, with the final hour allowing up to 2 ounces of clear liquid. Some centers will ask TN-EG or TN-EGD to have an 8 h fast period, especially if there is a concern for gastroparesis. If any meals are taken on the day of the procedure it is recommended to be low fat as well. NPO guidelines are not for patient safety as the patient is awake, they are to minimize potential emesis and optimize gastric visualization. This would indicate that NPO violations are permissible if the provider is comfortable.

On the day of the procedure visit, often child life specialists, if available, will meet with the patient and family before the procedure to re-explain each step and address any questions. If not available, this is often done by a nurse or provider. Once the patient and their parents are comfortable with the procedure, a nurse will often apply oxymetazoline to the nostrils to help decrease nasal congestion and open the nasal passages. This may occur in a counseling room or in the room of the procedure due to its duration of action. Patients with allergic rhinitis are strongly advised to take their allergy medications consistently for two weeks leading up to the procedure.

After applying oxymetazoline, 4% lidocaine spray (40 mg/ml) or 2% gel (20 mg/ml) is used in the nose and back of the throat to reduce discomfort. There is a wide dose range and response curve, but most commonly centers report max dose of mucosal analgesia at 4 mg/kg/dose not to exceed 300 mg. Sometimes other regimens are used (36, 37). It should be customized per site and patient for optimized experience. A nasal atomizer is used to administer the liquid spray vs. a syringe for the gel. The author's most commonly recommended dose for syringe-based atomization (4% lidocaine) is 1 ml in each nostril and 1 ml in the back of the throat. (Total dose 3 ml, 120 mg) Care should be taken to assure proper atomization/mist occurs. If additional dosing is needed for analgesia most cases can receive additional lidocaine. If a metered spray dosing device is used (MADomizer/Telefex), 0.5 ml in each nostril and 0.5 ml in the back of the throat seems to provide optimal analgesia. Each spray of this device provides 0.1 ml of liquid and provides more reliable atomization. If TN-EG/EGD is performed, commonly a larger dose is used to enhance the duration of the medication as the procedure takes longer. This dosing is customized per patient.

Lidocaine has a bitter taste, so in pediatric patients, an allergen-free lollipop is often provided to help mask the flavor. It also seems to help alleviate anxiety. Sucking on the lollipop, even a red color, does not interfere with the visualization of the upper GI anatomy. The lidocaine medication usually will last 5–10 min before it starts to wear off and giving just prior to the procedure with limited break is recommended. If the procedure is expected to be longer, such as with TN-EGD, a larger dose or re-dosing of lidocaine during the procedure may be considered, following dosing guidelines to ensure optimal patient comfort. The authors commonly use 10 sprays of the MADomizer device (1 ml) in each nostril and 10 sprays in the back of the throat for TN-EGD as an example. This is a total dose of 3 ml (120 mg) of 4% lidocaine.

Once the patient reports feeling a “lump” in their throat, typically within 60 s, two positioning options are provided. Sedation-free TNE can be performed either in a seated upright position or in the left lateral decubitus position. The left lateral decubitus position may be more comfortable for younger children who are too short to sit comfortably in an adult chair, or for those with a history of orthostasis. Patients are given the option to wear virtual reality (VR) goggles during the procedure to help distract them from discomfort and anxiety (18, 26). Many patients find this helpful, and it is recommended especially during their first endoscopy. It is recommended to have the VR system set up and calibrated prior to administering the lidocaine due to the duration of the medication and technical issues of VR that can occur.

Just prior to starting the procedure, it is recommended to have the patient blow their nose, especially if gel-based lidocaine is used. The flexible endoscope is then lubricated before being inserted into the nasal cavity. A water-based lubricant, lidocaine jelly, or silicone spray can be used. Care should be taken to minimize contact between the scope and the nasal turbinate or adenoid tissue, as this can cause significant discomfort. Intubating along the inferior turbinate will allow for more significant comfort than the middle turbinate in most cases, but the passage may be narrower. The endoscopist should be able to intubate either nostril as often one turbinate passage is less swollen than the other. To prevent choking and aspiration, it is also advised not to use air or water while the scope is in the nasopharynx or oropharynx. If the camera lens becomes obstructed by nasal secretions, the patient may be asked to sniff, which often clears the lens. A swallow prior to esophageal intubation while the scope is in the back of the pharynx can often clear the endoscope lens if stuck nasal secretions are obscuring the view in the pharynx. The patient is then asked to swallow to facilitate esophageal intubation when it's time to intubate the upper esophageal sphincter.

**Figure 1:**
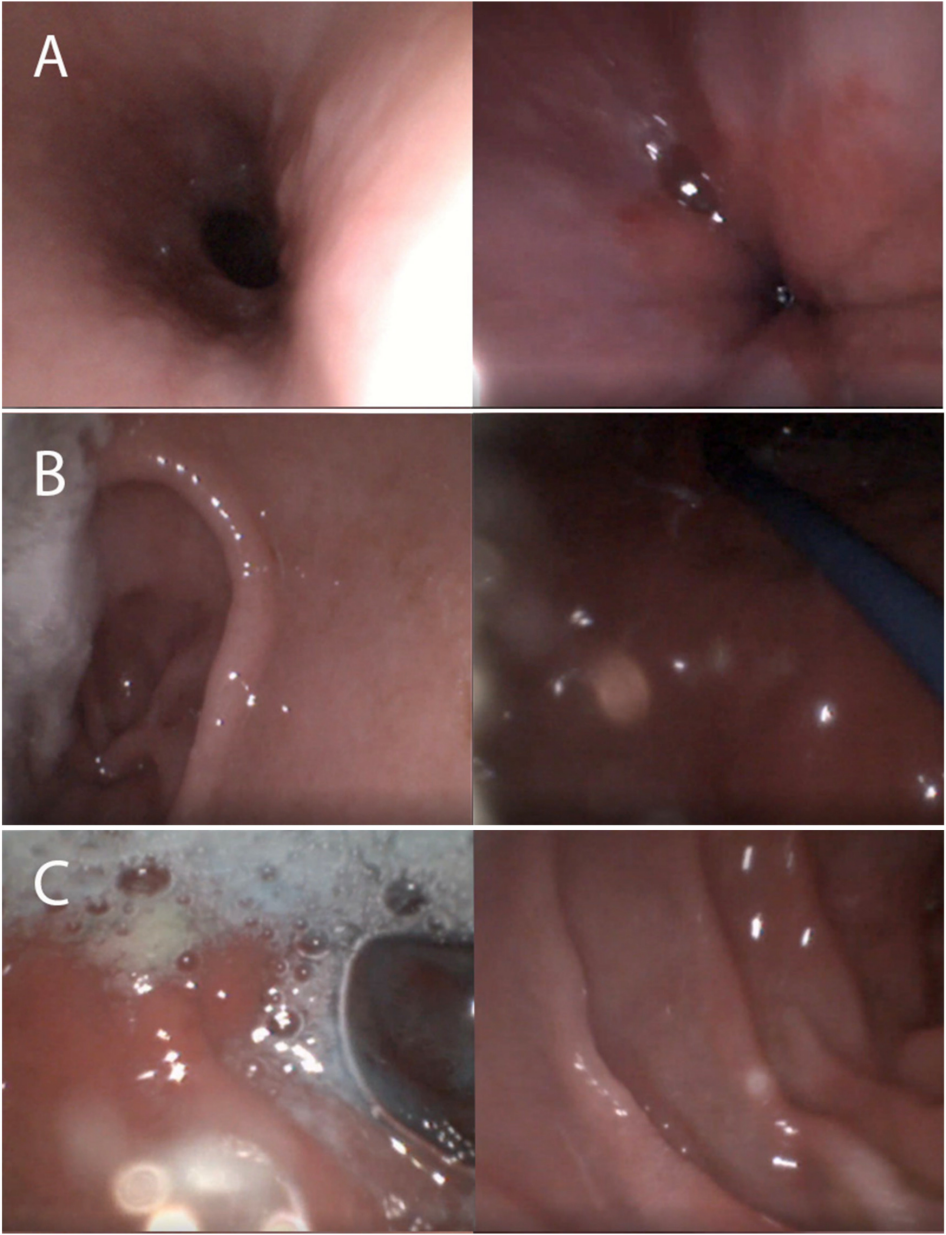
Images from transnasal esophagogastroduodenoscopy. **(A)** Esophagus/LES **(B)** Antrum/Fundus **(C)** Bulb/Duodenum.

Once the scope is in the esophagus, the technique is similar to that of a standard sedated endoscopy of the gastrointestinal tract, esophagogastroduodenoscopy (EGD), but much finer movements are needed due to the outer diameter of an ultra-slim endoscope. Images from a diagnostic TN-EGD are pictured (Figure 1). Introducing too much air into the stomach can cause discomfort and nausea, so patients are encouraged to burp during the procedure to release some of the air. Water instilled in the esophagus is often comforting to the patient during the procedure, but letting the patient know it is being given is often helpful. Excessive water instillation can lead to vomiting. If attempting to perform TN-EGD in the left lateral decubitus position, it may make it easier to identify the pylorus, as the gastric secretions naturally move away from it. If the procedure is prolonged beyond 10 min, an additional dose of lidocaine can be given alongside the endoscope using the atomization device. Calculation of max dosing is recommended. Additionally, re-lubrication of the scope is recommended as the scope gets further into the gastrointestinal tract. Finer movements than those typically used with a larger-diameter endoscope are required. This may take some practice. Previous studies have documented the learning curve when learning TN-EGD with ultra-slim endoscopes (13, 26).

When removing the scope, the tip should remain centered in the lumen and be withdrawn slowly. Due to ongoing peristalsis and diminished air use in an awake patient, esophageal and gastric views can be obscured, but with expertise on the handling the ultra-slim endoscope and timing with a peristalsis and air insufflation, adequate visualization can be achieved. After the procedure, the patient is offered a popsicle or cold juice to soothe their throat. Once the lidocaine completely wears off, typically within 15–20 min, patients can resume eating and drinking as usual.

Sedation-free TNE is generally well-tolerated, with minimal complaints or adverse effects: emesis in 2.7%, spit-up in 3.1%, epistaxis in 3.7%, and nausea in 0.3% of patients (18, 31). Some patients may experience a brief syncopal or pre-syncopal episode (18). For these individuals, the left lateral decubitus position may be more suitable than the upright seated position during sedation-free TNE. It has also been reported to have significant fewer adverse events than sedated endoscopy of the gastrointestinal tract (31).

## Establishing and growing a sedation-free endoscopy program

Establishing an unsedated transnasal endoscopy program is a unique undertaking with unique challenges. As with the initiation of any disruptive technology, there will be resistance from administration, staff, and maybe even colleagues. Having a business plan and a vision will help overcome many of these obstacles. Being familiar with the appropriate procedural billing codes is also of utmost importance (36). The first assumption is that this will take away from standard endoscopy which could disrupt revenue streams and reimbursement. The goal of such a program would not be to replace all endoscopy procedures but rather supplement the existing program. It is critical that people understand that there are some limitations to what can be done with an ultra slim gastroscope. Unsedated endoscopy with an ultra slim scope is excellent for surveillance procedures and interventions that have minimal pain. It is also useful when evaluating patients who have a high-risk of anesthesia or who have low risk conditions with low likelihood of significant findings requirement interventions such as reflux, vomiting, or abdominal pain. With experience, doing TN-EGD could also be used for celiac disease evaluation with abnormal IgA TTG, but it takes expertise in using an ultra-slim endoscope in a sedation-free environment (18, 38). An ultra slim endoscope could also be used under anesthesia for more invasive procedures, but this will be addressed later. Limitations of sedation-free TNE do exist and these are usually related to the ultra slim technology itself: feel of the scope itself, the diameter of the working channel, and the lighting that will be less than a larger diameter endoscope (10).

There are four key areas when discussing with a hospital business leader and trying to form a business plan for a sedation-free endoscopy program. First and foremost, it should be stressed it is optimal for patient care to avoid anesthesia and unnecessary risks in the appropriate patient populations (31). A second attractive feature of sedation-free endoscopy is that it does not require an operating room or a procedure room that could now be used for a different patient who requires anesthesia. In business planning it should be stressed that TNE does not replace sedated procedures but rather adds an additional tool and possibility of increased access and availability with potential for increased additive endoscopy (30). Third, having the option of being able to perform these procedures at your institution will likely bring in more referrals that otherwise may not have seen (18). Referrals can come through marketing, pediatricians referring to your institution and ultimately word of mouth from your patients. With the new American College of Gastroenterology (ACG) guidelines suggesting in pediatric populations TNE should primarily be used to surveil EoE and sedated EGD to be used only if TNE is not tolerated, this is even more important (39). It would seem advantageous to have a program established instead of trying “to catch up” and lose patients to other centers. Further expansion to other populations in the future (i.e., variceal surveillance, celiac monitoring, reflux esophagitis or *H pylori*) can further increase procedural volume to a center. Finally, the endoscopy access, diagnostic efficiency, and customer service is important to consider. TNE allows the possibility of quicker and more efficient diagnoses for patients without the burden on the family, community, and health system (30).

Although many think TNE will not be embraced by patients, the authors have found surprisingly, that marketing this technique to families has not been a challenge. When a provider and their colleagues are confident about the procedure being performed, many families embrace it and are excited about the possibility of avoiding anesthesia. It lowers the risk of adverse events and possibly allows for more frequent surveillance especially in cases of the eosinophilic esophagitis where frequent scopes are necessary to monitor disease progression or remission (18, 31). The patients, albeit less excited than their parents, still show enthusiasm with the thought of VR goggles being used as a distraction as well as the use of lidocaine to help lessen any discomfort (35). Many older patients like the idea of being able to walk right out after the procedure without having the lingering effects of an anesthetic (35). Patients should be aware that because they decide to try unsedated endoscopy does not mean that they cannot go back to a traditional sedated endoscopy if they find the procedure unpleasant. The authors would not advocate asking them “what did they think” immediately after the procedure but rather give them a few days to reflect as the immediate memory of any discomfort could bias their opinion. Also, in preparation of the procedure, families should tell their child this will be different than previous endoscopies. It is to no one's benefit to have the child be surprised when they get there and find out they are not receiving anesthesia. Using a saline nasal spray prior to the procedure could help accustom the child to something going up their nose. Having a child life specialist present to calm any anxiety might also be beneficial if resources allow for this intervention. There are video resources available online so that children can see the procedure being performed prior to coming for the actual visit.

## Training in sedation-free TNE

The logical question is how to prepare or train the endoscopist using a new slimmer device, especially on a patient who is fully aware of everything happening in the room and an often-anxious parent watching. There are two types of endoscopists that need to be trained in sedation-free endoscopy: the usual trainee and the new endoscopist who may be confident in general endoscopic concepts. The latter would be a practicing gastroenterologist considered proficient in routine diagnostic or therapeutic endoscopy. Finding a mentor who can train either endoscopist type is important, though finding one proficient in innovative techniques could be difficult. Though studies have documented self-training success in the adult and pediatric population, expert sedation-free endoscopists may help prevent the pitfalls that independently-performing endoscopists fall into (13, 14, 36). Many of the authors find training fellows or advance practice providers may be easier than training expert trained endoscopists as they are more open to new techniques and don't have “muscle memory” of navigating larger diameter devices. The practicing endoscopist may even require more training and practice in TNE than a novice endoscopist.

Transnasal endoscopy is an upper gastrointestinal procedure like any transoral endoscopy. It is commonly credentialed in a hospital in the usual sedated environment but doing it on awake patients may require enhanced skill navigating unfamiliar anatomy. Additional credentialing may need to be filed with the hospital for the physician or the nursing team. The mentor can help teach this and the idiosyncrasies associated with sedation-free TNE. They may offer training concepts that may not be readily apparent to a new ultra-slim endoscopists. They may also offer advice on various available devices and pros/cons of each. The device industry also does have training courses available on using such devices in the gastrointestinal tract. A mentor could be another gastroenterologist trained in TNE. A mentor could also be another subspecialist in nasal anatomy (i.e., Otolaryngology or Pulmonology). These specialists may teach skills that focus on different techniques to accomplish a complete nasal or pharyngeal exam as compared to using it as passage to get to the gastrointestinal tract. This potentially could confuse a gastroenterologist or prolong a procedure that could be shorter. Appropriate mentor-mentee pairing is of utmost importance.

Prior to performing a TNE on a patient, endoscopists should establish familiarity with both the device and nasal anatomy to optimize the patient experience. Practice is critically important. Training models at local institutions or national hands-on courses can aid with learning curves as endoscopists approach the procedure on actual patients.

Parents and patients want an endoscopist who is competent and confident. Volume is important when building a program, with considerations for adequate volume both to establish and maintain procedural competency over time. The authors have generally found that general proficiency typically develops after 5–10 procedures while confidence may take up to 20–30 + procedures, depending on the endoscopist. As an endoscopist approaches >100, confidence in TNE can be akin to that of traditional sedated endoscopy. It is important to persevere through the initial few procedures where the endoscopist is nervous and the patient experience may not be perfect. An optimal strategy to establish initial competency would be to schedule 3–4 patients per session with 2–3 sessions per month initially. This is also demonstrated in the literature when self-training documented success at 60–70 TNE performed within 6–7 months (13).

Patient selection, as noted above, is also especially important during training. The authors recommend that when selecting the first few patients (Total # 5–10), it might be best to start with older, calmer teens and preteens rather than school aged patients. An obviously anxious patient may not be ideal, especially when first learning. Sometimes patients 8–10 years of age will be easier to perform the procedure better than teens, but the anatomy may be smaller which may make it hard to navigate the nasal anatomy for a new nasal endoscopist. Using previous patients who have had TNE done before and have tolerated the previous procedure well may be ideal (33).

During training and depending on the center, parents are often in the room while the unsedated endoscopy is being performed. Some centers may consider asking the parents to step out during training to avoid increased stress or too many people in the room. Also, depending on the comfort of the endoscopist, the parents might be told that findings will be explained at the end rather than during the procedure. TNE, as the authors have noted, is true multi-tasking of procedure management, room management, patient management, and family management. The TNE endoscopist will likely learn one skill at a time and slowly be able to do all at the same time. The mentor will help with that. For example, managing the family and giving clear guidelines of what to do may also be important, as the parent may increase the stress of the trainee or the patient. It may be important to let the family know that the endoscopy images are magnified on the screen (i.e., a drop of blood from a biopsy might appear to be a large GI hemorrhage). Also important is an avoidance of medical jargon and phrases such as “my goodness, that looks like a lot of blood” in front of the unsedated patient who has a scope inside them. The language used when training a fellow in this procedure should be positive and suggestive. Sometimes non-verbal education, such as pointing on the screen or usual virtual guidance from a remote camera site can be helpful. Offer guidance and avoid phrases that would undermine the patient's confidence in the trainee. Finally, the patient can participate in the training and can speak. This is especially true if the patient has had a previous TNE done. Some may ask the patient if there is discomfort during the procedure because sometimes simply repositioning the head or raising my hand might relieve torque on the scope and make the procedure more comfortable for everyone. However, teaching the trainee to avoid excess questioning about the procedure while performing the procedure is important. It can increase the patient's anxiety and creates additional discomfort. TNE is still a medical procedure. Teaching how to be reassuring to the patient and trainee while understanding what to look for and correct errors in technique is of utmost importance.

## Section 3: advanced applications of ultra-slim endoscopy in pediatric GI: sedated and select unsedated uses

The clinical utility of ultra slim endoscopes has expanded significantly, encompassing a range of diagnostic and therapeutic interventions beyond traditional unsedated transnasal endoscopy. In pediatric gastroenterology, these endoscopes offer unique advantages in scenarios where standard caliber instruments may be limited by patient size, anatomy, or procedural complexity. This section reviews advanced applications of ultra slim endoscopy, primarily in sedated environments but also including select unsedated techniques. Technical considerations, device compatibility, and procedural workflows are outlined to support integration into specialized pediatric endoscopic practice.

## Esophageal strictures

Esophageal strictures or stenoses are serious problems in children that can occur for several reasons. In older children, esophageal strictures may develop due to eosinophilic esophagitis, whereas in infants and young children, they often result from Esophageal Atresia (EA) with or without tracheoesophageal fistula (TEF). EA is the most common congenital anomaly of the esophagus, with an overall incidence of one in every 2,500 to 4,500 live births (40, 41). The rate of esophageal strictures following EA repair is underreported, with a reported incidence ranging from as low as 9% to as high as 80% (42–47). Endoscopy plays a critical role in both the surveillance and treatment of children with EA (25, 48). While an adult gastroscope can be used safely in children weighing more than 10 kg, there are many reasons and benefits to using a slim gastroscope (49).

### Assessment of stricture

When assessing an esophageal stricture, several factors should be considered. First, it is crucial to determine the diameter and length of the stricture. Additionally, evaluating the quality of the tissue above and below the stricture can help identify the underlying etiology (e.g., peptic or eosinophilic esophagitis). If there is no recent esophagram to guide you, using a slim or ultra-slim gastroscope can be invaluable. Its smaller diameter may allow it to pass through tighter strictures, enabling a more accurate assessment of both stricture length and diameter. It is also essential to know the outer diameter of the scope you are using, as this helps approximate the stricture diameter. If a slim gastroscope cannot pass across the stricture, and if the patient has a gastrostomy tube (may be present in those with esophageal atresia), passing the slim gastroscope through the gastrostomy stoma and viewing the esophagus retrograde can be extremely helpful for evaluating the tissue below the stricture. Reusable gastroscopes typically require a gastrostomy tube stoma to be at least 16 Fr, so dilation of the stoma may be necessary prior to passage. Alternatively, the reported above single-use gastroscope or off-label flexible endoscopes (i.e., bronchoscopes, cystoscopes) can often pass through a 12 Fr gastrostomy stoma.

### Esophageal stricture dilation

The foundation of esophageal stricture treatment is dilation. The goal of esophageal dilation is to increase the luminal diameter of the esophagus while alleviating dysphagia. This is achieved through circumferential stretching and splitting of the scar tissue within the stricture (42, 50). Though there are reports of adult sedation-free stricture dilation, it is most often performed with sedation (51). The most common approach to esophageal dilation is balloon dilation, which delivers equal radial force across the entire length of the stricture. Through-the-scope (TTS) balloon dilation allows the endoscopist to directly visualize the stricture during and immediately after dilation. However, TTS balloon dilation requires a standard adult sizes gastroscope with a minimum working channel diameter of 2.8 mm, and currently, there are no commercially available balloons designed for use in a slim gastroscope. In children weighing less than 10 kg, passing an adult gastroscope to perform TTS dilation can be challenging. Even if scope passage is possible, it is important to note that the scope can cause significant compression of the airway, making ventilation difficult. This is a critical consideration for any child under 10 kg, but especially for those with EA, who often have some degree of tracheomalacia. Even in EA patients over 10 kg with severe tracheomalacia, passing an adult gastroscope may compromise ventilation. In contrast, dilating with a slim gastroscope in young children is often feasible and generally exerts less compression on the airway.

There are two approaches to dilation with a slim gastroscope. The first involves passing a balloon over a guidewire under fluoroscopic guidance. This is done by introducing a 0.035-in guidewire through the endoscope working channel across the stricture, then removing the scope while leaving the wire in place, and finally sliding the balloon over the wire and positioning it under fluoroscopic visualization (42, 52).The second approach is to pass the balloon alongside the slim endoscope and advance it across the stricture under direct endoscopic visualization. In this technique, the guidewire remains within the balloon and can be advanced across the stricture under endoscopic guidance. This approach can be more difficult in tighter strictures, as the scope may push the balloon to one side, but it allows the endoscopist to minimize or avoid fluoroscopy. [2,10] Regardless of the approach, a balloon should never be passed blindly through a stricture if the scope cannot traverse it first, unless you have prior knowledge of the tissue beyond the stricture. The guidewire should be advanced into the stomach to ensure a safe path, thus preventing accidental perforation by the dilator tip.

### Adjunct treatment for strictures with a slim gastroscope

Intralesional steroid injection (ISI) is typically used alongside dilation to facilitate a larger post-dilation esophageal stricture diameter (53). The proposed mechanism of ISI is to locally inhibit the inflammatory response that promotes collagen formation and scarring within a stricture (42, 52, 53). ISI is administered via a sclerotherapy needle in 0.1–0.2 ml aliquots. Four-quadrant injections are common; however, if the scar tissue is uneven, a greater volume of steroid can be injected into the areas with denser scar tissue. The dose of triamcinolone acetonide used is 1–2 mg/kg per dose, up to a maximum of 40 mg (42). Several endoscopic injection needles are commercially available that can pass through a slim gastroscope.

Endoscopic electrocautery incisional therapy (EIT) involves applying electrocautery with a needle knife to create small incisions in the thickest areas of scar tissue, forming preferential weak points. After these incisions are made, balloon dilation can be performed to preferentially expand the areas weakened by the incisions. This technique is typically more effective for strictures less than 1 cm in length. In a pediatric study of 58 patients, EIT successfully resolved the stricture in 76% of patients over a 2-year follow-up (54). While most commercial knives require a 2.8 mm channel, there is a commercially available needle knife that can pass through a slim gastroscope (Table 2). For narrow strictures in older patients, EIT with the slim gastroscope is well-tolerated and may allow for better maneuverability and incision placement. Using a slim gastroscope is also preferable when performing EIT in children under 10 kg. Note that these considerations apply to reusable slim gastroscopes, as the current single-use scopes are not cleared for electrocautery/active therapeutic indications.

## Surveillance of esophageal varices

Esophageal varices (EV) can be a life-threatening complication of patients with portal hypertension. Often, patients require regular endoscopic evaluation and monitoring, though there are no clear clinical guidelines for screening in pediatric patients (6, 55). Ultra-slim endoscopes have been used via a transnasal route to identify esophageal varices in adults (6, 56). Endoscopic visualization of varices is of utmost importance. A recent pilot-study (submitted for publication) evaluated the use of ultra-slim gastroscopes for accurate identification of esophageal varices in pediatric patients, corroborating findings with standard of care larger oral gastroscopes.

In 10 subjects who underwent routine sedated surveillance endoscopy for EV, esophagoscopy with both a 3.5 mm outer diameter single-use endoscope and a standard larger diameter (>8.6 mm) gastroscope in the same session showed 100% visual concordance for the presence or lack of EV, normal mucosa, and esophagitis. In 7/8 (87.5%) of patients who had identifiable varices, visual grade matched between both devices.

That study also demonstrated the potential for a pediatric hepatologist to consider the use of TNE for EV evaluation in a lower cost, lower acuity setting such as TNE reported in adults (6, 56). Further research is needed however as the subjects were supine and sedated. It did however demonstrate that ultra-slim endoscopes could visualize varices similarly to larger caliber endoscopes. If ultra-slim endoscopes in pediatrics demonstrate similar results to adult studies, the potential use of ultra-slim gastroscopes for secondary surveillance may allow for avoidance of unnecessary anesthetic events as seen in the cohort where 70% of patients did not ultimately require therapeutic intervention (56, 57). Patients with no history of high-grade varices necessitating endoscopic therapy may be ideal patients to undergo surveillance with sedation free TNE. A second phase of that study, examining use of sedation-free TNE for secondary surveillance of EV is currently planned.

Given its size, ultra-slim endoscopy is limited in its therapeutic capabilities for bleeding esophageal varices. There is no known banding device that is currently compatible (10, 58). Sclerotherapy with injection needles that fit within the 2.0 mm working channel (Table 2) is a viable option in small patients <10 kg when performed under general anesthesia. Sclerosants include sodium morrhuate (2.5%–5%), sodium tetradecyl sulfate (1%-3%), and ethanolamine oleate. These agents act as tissue irritants that cause localized thrombosis and endothelial damage, ultimately leading to fibrosis and obliteration of the bleeding vessel.

Injection amounts are typically small aliquot volume ranging from 0.3–1 ml per injection site (59). Gastric varices can be injected with cyanoacrylate glue in similar sized aliquots for more effective hemostasis than endoscopic band ligation. This glue polymerizes immediately with blood to produce vascular obliteration and mixing 1:1 with lipiodol can facilitate glue administration through the endoscope (60). Gastrointestinal lumen and lesion visualization is of utmost importance, especially if a therapeutic procedure such as injection is considered.

## Direct placement of nasal enteral feeding tube

Enteric feeding tubes are a mainstay of nutritional management for pediatric patients. Placement of enteric tubes, including nasogastric (NG) or nasojejunal (NJ) tubes can be facilitated by fluoroscopy or endoscopy. With adequate expertise, endoscopic placement limits radiation exposure and can decrease procedure time due to direct luminal visualization, and allow for bedside placement (61, 62). Direct visualization nasal enteric tube placement may be more advantageous in cases of esophageal stricture, as detailed above, and severe mucosal injury where there is higher risk of perforation. Transnasal placement of an NG or NJ tube involves advancement of a guidewire through a gastroscope, withdrawal of the gastroscope leaving the guidewire in place, and then placement of the enteric tube over the guidewire (63). Direct transnasal placement potentially could be easier than facilitated transoral endoscopy pulling a transnasal enteral tube into the digestive tract.

## Bedside diagnostic and therapeutic endoscopy

Bedside endoscopy can be used in pediatric patients for diagnostic purposes or for therapeutic interventions such as percutaneous endoscopic gastrostomy (PEG) placement (61). One of the main barriers to performing bedside procedures includes physical space limitations for endoscopy equipment. Endoscopy “towers” with processors, monitors, and equipment storage are bulky and cumbersome to move. These may be especially difficulty to maneuver in small hospital rooms or intensive care units, where critically ill patients have rooms already filled with equipment such as medication pumps, dialysis machines, and extracorporeal membrane oxygenation (ECMO) circuits. Customized mobile endoscopy carts with attached monitors, akin to “computer-on-wheels” oft-seen in hospitals, provide increased mobility and flexibility to perform bedside procedures. These carts can be built with smaller profiles without dedicated equipment for water, suction, or air, using portable sterile water containers and in-wall suction and air in hospital rooms.

Patients may also have significant risks related to anesthesia that pose a barrier to performance of conventional sedated endoscopy. Sedation-free endoscopy at the bedside allows for increased diagnostic and therapeutic options for patients. Diagnostic endoscopy through a stoma or per-rectum may be more easily performed and better-tolerated with an ultra slim flexible gastroscope. Additionally, ultra slim endoscopy has been used by the authors at the bedside in unsedated patients for intra-pyloric botulinum injection.

Sedation-free transnasal bedside endoscopy has also been noted by the authors to be helpful in cases with diagnostic uncertainty, including persistent globus, retained foreign body, and suspected gastrointestinal bleeding. Not uncommonly, gastroenterologists are consulted on patients with persistent globus where radiographs do not visualize radiopaque foreign bodies or fluoroscopic studies do not show a clear filling defect. In these scenarios, bedside endoscopic evaluation in the emergency department or inpatient setting can be a useful screening test to spare admission, prolonged hospital stays, or unnecessary sedation. If foreign bodies are visualized in the gastrointestinal tract, utmost caution should be taken for any attempted removal, as patients may not have adequate airway protection and are high risk for aspiration.

Similarly, patients may present with signs of upper gastrointestinal bleeding such as coffee-ground emesis or melena, though have reassuring hemoglobin and/or hemodynamic stability. Gastroenterologists may choose to defer endoscopic evaluation, opting to monitor a patient over time. Transnasal or trans-stomal endoscopy, when applicable, may be a screening option that assesses for lesions that need further therapeutic intervention such as electrocautery or clip placement. Though these bedside procedures would be used primarily for diagnostic purposes, temporizing hemostasis could be achieved with injection of epinephrine or side-by-side hemospray (channel required 2.8), until a therapeutic procedure under anesthesia is performed. Theoretically, therapy of variceal bleeding with sclerotherapy could be performed in the unsedated patient, however clinically this is not recommended due to the risk and possible need for advanced intervention with a larger scope.

## Transgastrostomy (trans-stomal) upper endoscopy *via* gastric stoma

A gastrostomy site, as well as other gastrointestinal stomas, can provide easy endoscopic access to the upper gastrointestinal tract for visualization and biopsy (29). Since entry through the gastrostomy avoids the stimulation of the posterior pharynx, the procedure can often be performed in a cooperative patient, with or without topical stomal analgesics. A slim endoscope (5.4 mm) can easily pass through a gastrostomy site with a 16 French or larger gastrostomy tube (GT). Some 14 French GT sites are also adequate. Narrow stoma sites can be dilated to with Hegar or anal dilators, but this process can be painful and may require anesthesia. Utilizing a slimmer scope (3.5 mm or less), such as off-label flexible non-gastrointestinal endoscopes (bronchoscope, cystoscopes, etc) or the recently released single-use, ultra-slim gastroscope, is another method to access the upper GI tract through a smaller stoma without the need for dilation. These endoscopes are not officially indicated for trans-stomal endoscopy. Bronchoscopes also have several limitations including their fragility, small biopsy channel, and restricted ability to clear the gastrointestinal secretions and insufflate. The lesser field of view (FOV) and dimmer light intensity of both the single-use gastroscopes and the very-slim non-gastrointestinal flexible endoscopes can also make viewing in the stomach more difficult. Specifications can be found in Table 1.

Transgastrostomy endoscopy is not a part of training at most fellowship programs but is routinely performed at many institutions. As with unsedated TNE, patient selection is important. A child that can remain relatively still is the best candidate for the procedure. Even if anesthesia is required, it still may be preferable to utilize the gastrostomy for an upper endoscopy in certain patients with a difficult upper airway to minimize stimulation and limit sedation. As noted above, the gastrostomy also may be the preferred access point if the patient is undergoing a retrograde esophageal dilatation or gastrojejunostomy tube placement. If no sedation is used, only 4–6 h of fasting is required to ensure that the stomach is empty prior to the procedure on a liquid diet. A longer fasting time of at least 6–8 for solid food is recommended. For unsedated procedures, the endoscopy team should have a child life and/or the parent at bedside. A device such as VR noted above or video/music can provide distraction. Transgastrostomy endoscopy is usually performed with the patient in the supine position, lateral, or a sitting position. Successfully execution of this procedure requires a familiarity with the use of a slim scope as well as an understanding of how to access the pylorus and esophagus given the altered scope orientation from the gastrostomy entry point.

**Figure 2:**
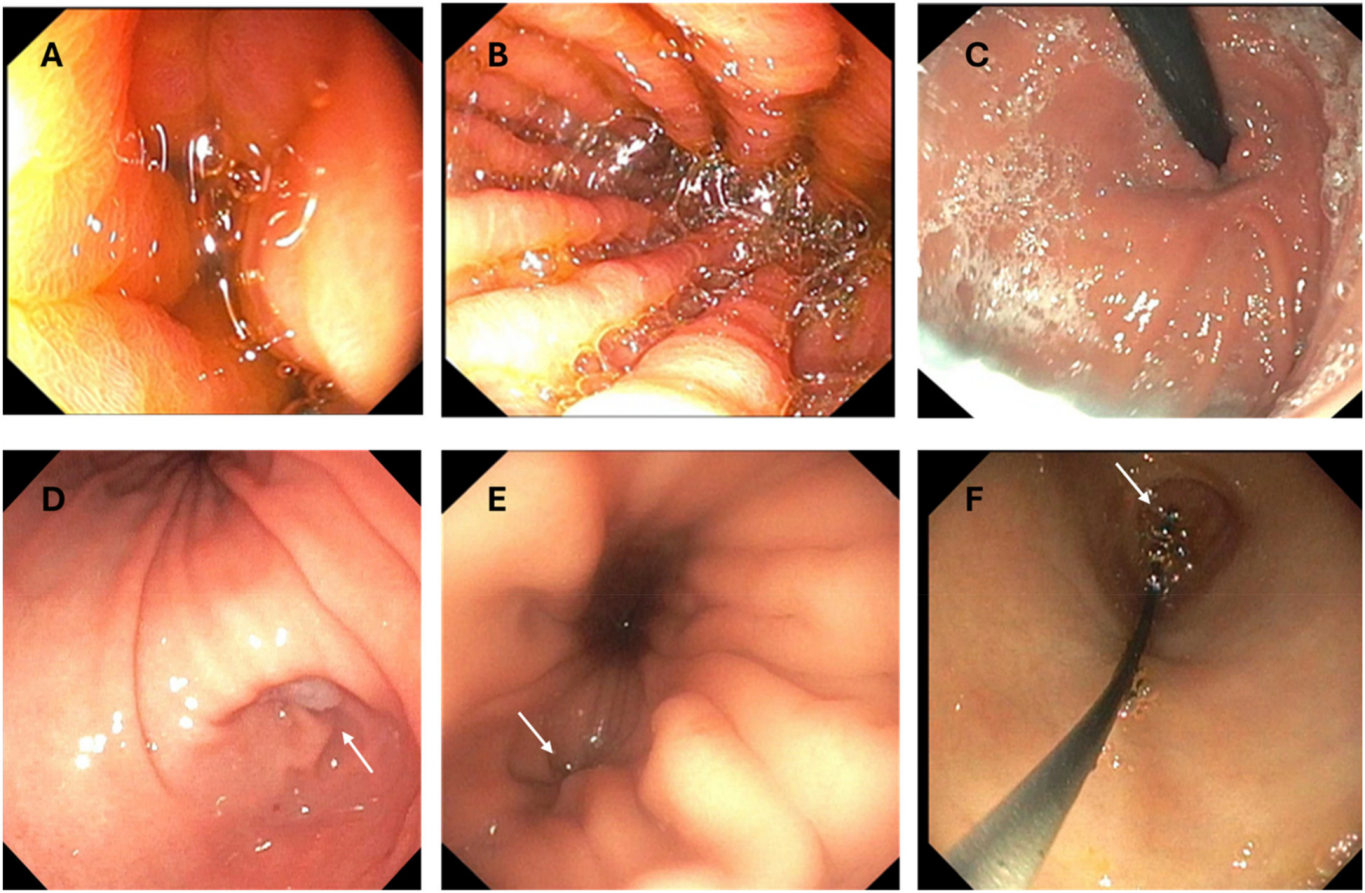
Images from transgastrostomy endoscopy. **(A)** Image after initial insertion without insufflation **(B)** Gastric body after insufflation **(C)** Retroflex view of the gastric stoma **(D)** Gastric body and antrum with arrow indicating pylorus **(E)** Arrow pointing to GE junction in gastric cardia **(F)** Glidewire exiting the pylorus (arrow) during scope withdrawal during a GJT placement procedure.

When the scope is initially introduced after GT removal, the stomach is collapsed, and insufflation is required for complete visualization (Figure 2A). To improve insufflation in patients with a larger stoma (relative to the diameter of the endoscope) a gauze or a finger can be used to plug or compress the stoma opening. Excessive insufflation can result in the patient burping or retching. In general, using the minimum amount of insufflation in an unsedated patient is the rule. Avoiding insertion of excessive looped scope in the stomach is also imperative for patient comfort.

Determining the location of the pylorus and the gastroesophageal (GE) junction can be a challenge. The pylorus can be very close to the gastrostomy stoma in some patients. Insufflation can help identify the landmarks including the body of the stomach (marked by rugae), the antrum (smooth), and the incisura separating the two (Figure 2D). If it is difficult to differentiate the body from the antrum after insufflation, the scope should be oriented on the outside of the body so that the tip is directed at either the pylorus (to the patient's right) or the esophagus (cephalad) without any significant tip manipulation. An assistant can assist in stabilizing the shaft outside the body (retrograde movements) while fine movements are performed by the endoscopist using the endoscope dials.

The esophageal junction is usually closed and located near the cardia of the stomach and slightly lateral to the gastric lumen (Figure 2E). Sometimes the GE junction is marked by a small dimple or irregularity in the gastric folds, but it can be difficult to visualize. If the patient can sip a colored or carbonated liquid, the opening may be more easily identified. Intubating the esophagus is the most challenging part of transgastrostomy endoscopy given the eccentric location of the GE junction and usually requires some manipulation of the scope tip. Once in the distal esophagus, care should be taken not to advance close to or beyond the upper esophageal sphincter which could stimulate gagging. This is usually the most stimulating part of the transtomal procedure and additional insufflation in the esophagus is usually the most uncomfortable. Working quickly is important. An assistant stabilizing the shaft during awake transtomal esophagoscopy can be of utmost importance to avoid inadvertent scope displacement into the stomach. Negotiating the small bowel relies more on scope tip manipulation than torque. It may be helpful in some situations to have an assistant hold the scope while the provider manipulates the scope dials to identify the lumen and advance the scope.

Most children tolerate unsedated transgastrostomy endoscopy well but there can be some discomfort related to scope passage through the stoma, insufflation resulting in gaseous distension and burping, stretching of the small bowel during deep advancement in the intestine, and gagging if the scope is advanced into the proximal esophagus. These symptoms can result in procedure termination in some patients. Otherwise, the risks and benefits are the same as conventional endoscopy.

## Transgastrostomy gastrojejunostomy tube (GJT) placement

Transgastrostomy GJT placement can be performed without anesthesia in a cooperative patient in an endoscopy unit or at the bedside (61, 64). Although interventional radiology is the mainstay for GJT placements, endoscopic placement has been shown in small studies to be safe and effective and limits exposure to radiation (64, 65). Added benefits for the endoscopic GJT technique is that the upper gastrointestinal tract can be fully visualized and biopsied during placement and that the procedure is performed by a GI team that is most familiar with the patient's tube and medical care. Complications from GJT placement are rare but include perforation, bleeding, infection and intussusception. The technique for endoscopically placing a GJT has been well described for both GT to GJT conversions and for primary GJT placements (61). In addition to a slim endoscope, fluoroscopy or a portable x-ray can be used to confirm tube placement. Other equipment needs include a stiff guidewire (0.89 mm in diameter) with soft straight or angled tip, the GJT, imaging contrast to confirm placement, and lubricant for the tube (Table 3).

**Table 3:** Practical considerations of transgastric GJ tube placement and billing codes and wRVU compensation for related procedures.

TRANSGASTRIC GJT PLACEMENT EQUIPMENT		
Slim endoscope (<5.5 mm diameter)		
Stiff glidewire with soft tip (0.035 inches)—primed with saline		
Water Based Lubricant		
1. Prior to procedure mix 2–3 ml lubricant with 10 ml of water in syringe and push through J port 2. Inject 2–3 ml into biopsy channel before wire insertion 3. Apply lubricant generously to the entire length of GJT prior to placement		
Gastrojejunal Tube		
Imaging Contrast Agent (Iohexol 300 mg/ml mixed ½ strength with water)		
Water to flush GJT after placement confirmed and to fill gastric balloon		
TRANSGASTRIC, TRANSNASAL, AND TRANSORAL CODES WITH WRVUs		
CPT	Procedure Description	wRVU
43,197/43,198	Esophagoscopy, flexible, transnasal, w/wo biopsy	1.52/1.82
43,235/43,239	Upper endoscopy w/wo biopsy	2.09/2.39
44,373	Small intestinal endoscopy with conversion of GT to GJT without fluoroscopy	3.39
49,446	Conversion of gastrostomy tube to GJT, percutaneous, under fluoroscopic guidance including contrast injection(s), image documentation and report	3.06
49,452	Replacement of GJT, percutaneous, under fluoroscopic guidance including contrast injection(s), image documentation and report	2.86
43,241	Esophagogastroduodenoscopy, flexible, transoral; with insertion of intraluminal tube or catheter	2.49
0654T	Esophagogastroduodenoscopy, flexible, transnasal; with insertion of intraluminal tube or catheter	Determined by Payer

For endoscopic GJT placements with fluoroscopy, code for an upper endoscopy (43,235/43,239) and a GJT placement with fluoroscopy (49,446/49,452). If the esophagus is not intubated, use reduced services modifier 52 for the endoscopy. Images with interpretation must be uploaded in the medical record for billing.

To place a GJT in a patient with mature gastrostomy site, the indwelling GT is removed, and the ultra-slim endoscope is advanced into the small bowel to a distance that is equal to the length of the GJT. Ensuring that the scope is oriented in the direction of the pylorus during advancement will prevent inadvertent looping in the stomach. A guidewire is then advanced through the scope into the small bowel. It is possible to advance the scope less than the length of the GJT if the wire can be advanced under direct visualization to the appropriate distance beyond the scope tip. To facilitate passage of the wire through the scope, it can be helpful to lubricate the biopsy channel with a water-based lubricant prior to wire insertion. Once the wire is in the small bowel, the scope is withdrawn while the wire is pushed forward by an assistant to prevent inadvertant wire removal. This is the most important part of the technique since adequate wire length must be in position after scope removal to facilitate appropriate GJT placement. As the scope tip moves back into the stomach, the endoscopist should see the wire exiting the pylorus without any loop in the stomach (Figure 2F). Then, as soon as the scope drops out of the stoma, the wire should be held in place by an assistant to prevent displacement.

Before tube insertion it is important to lubricate the jejunal lumen of the GJT. To do this, draw up 2–3 ml of water-based lubricant into a syringe followed by 10 ml of water. Shake the syringe vigorously and inject the mixture into the J channel. Generously lubricate the GJT and wire and then gently and slowly push the GJT over the wire through the gastric stoma while pointing the tube shaft toward the pylorus. Resistance is sometimes encountered as the tip of the tube meets the pyloric sphincter or as it encounters turns or folds in the small bowel. If there is resistance, pull the tube back a couple of centimeters and gently to work the tube back and forth till the resistance is overcome and advancement achieved. Pushing the tube when there is resistance could result in loop formation in the stomach which could predispose the tube to displacement. Fluoroscopy can be helpful if there is persistent resistance with tube advancement and to ascertain progress. Final position is confirmed by injecting contrast through the J port and obtaining an image with fluoroscopy or a portable x-ray. If bile can be vented from the J port, that is also a reassuring sign that the tube is in appropriate position. GJT replacements can usually be performed without endoscopy with an over the wire exchange if the appropriate position of the indwelling tube is confirmed using fluoroscopy with contrast injection.

Endoscopic primary GJT placements in a patient without a gastrostomy can be performed by creating a gastropexy (laparoscopic, endoscopic or fluoroscopic), followed by stoma creation with a dilator, and then advancement of the scope into the small bowel through the dilator sheath (66). The GJT can then be placed using same technique described above.

## Conclusion

Ultra-slim flexible endoscopes have advanced considerably since their development over 40 years ago. Though their original design was to navigate small anatomy their use in the pediatric gastrointestinal tract has enabled newer, more convenient, and more accessible diagnostic and therapeutic endoscopy. Various uses of such endoscopes in the pediatric world have been described and will continue to advance as the technology continues to evolve. Their use has potential in both a sedated and sedation-free environment.
